# The psychological impact of the COVID-19 pandemic and the role of resilience: cross cultural differences between Brazil, Italy, and the United States

**DOI:** 10.1186/s12889-023-16687-4

**Published:** 2023-11-17

**Authors:** Maria C. Quattropani, Marcus Levi Lopes Barbosa, Vittorio Lenzo, Keely Hope, Mary Ellen Toffle, Leonardo Gonçalves Gafforelli, Alberto Sardella, Kayleen Islam-Zwart

**Affiliations:** 1 Department of Educational Sciences, University of Catania, Via Biblioteca 4, 95124 Catania, Italy; 2 Universidade Feevale, RS-239, 2755, Vila Nova, Novo Hamburgo - RS 93525-075 Brazil; 3 School of Psychology, Professor, Eastern Washington University, 135 Martin Hall, Cheney, WA 99004 USA; 4 Department of Political and Juridical Sciences, University of Messina, Piazza XX Settembre, 4, 98122 Messina, Italy; 5 Department of Clinical and Experimental Medicine, University of Messina, Consolare Valeria St, 98125 Messina, Italy

**Keywords:** COVID-19, Clinical psychology, Resilience, Depression, PTSD

## Abstract

**Objective:**

Restrictive measures consequent to the COVID-19 pandemic have had a significant psychological impact on everyday life in the general population, even though differences between countries remain poorly investigated. The present study sought to examine the different psychological impacts and resilience of the pandemic among three of the most heavily hit countries: Brazil, Italy, and the United States.

**Methods:**

This cross-sectional study separately involved three national community populations, namely the Brazilian, the Italian, and the American population. Participants aged 18 years or older were recruited through a shared online survey. Participants self-completed the Connor-Davidson Resilience Scale (CD-RISC-10) and the Center for Epidemiological Studies-Depression Scale (CES-D); post-traumatic stress was additionally assessed using the Impact of Event Scale—Revised (IES-R). Three separate Analyses of Covariance (ANCOVA) were performed in order to investigate differences in the levels of resilience, post-traumatic stress, and depression among the three populations.

**Results:**

The study included in total 734 participants (mean age = 27.60 ± 11.69 years; 77% of females). Results of ANCOVA comparisons showed significant differences between the three groups in the variable measuring resilience, post-traumatic stress symptoms, and depression. As for resilience, results of post-hoc tests showed significant differences between the groups from Brazil and Italy and between the groups from Brazil and USA. As for the post-traumatic stress symptoms, results showed significant differences between the USA and Brazil groups and between the USA and Italy groups. As for the depression symptoms, results showed significant differences between the USA and Brazil groups.

**Conclusions:**

Overall, these findings may help to increase understanding of the psychological impact of COVID-19 in Brazil, Italy, and the USA. Interventions to prevent mental disorders among general populations should take into account these findings.

## Background

Since December 2019, when a series of cases with pneumonia of unknown etiology in the city of Wuhan was described [1], the coronavirus disease-2019 (COVID-19) has quickly spread worldwide. At the beginning, severe restrictive measures involved millions of people who were forced to stay at home. Despite the efforts of the health authorities in the COVID-19 hit countries, the number of confirmed cases has heavily increased in the world, with 6.952.522 deaths as of 26 July, 2023 [2]. While not everyone has contracted the COVID-19, most of us have experienced its psychological impact, together with a heavy economic burden and great financial losses along with social isolation resulting from the implementation of national lockdown [3].

In pursuing this question, a review and meta-analysis including 72.004 participants found the pandemic to have a small psychological impact, in spite of great heterogeneity across the studies [4]. One of the first nationwide studies involving a large sample of 52.730 Chinese participants found that about a third of them experienced severe psychological distress [5]. Another early nationwide study conducted in China found that the majority of participants experienced a moderate to severe psychological impact [6]. Although China was the first nation to be impacted, the COVID-19 rapidly circulated in further countries, such as Brazil, Italy, and the United States.

The burgeoning literature on the COVID-19 in Brazil has depicted a huge impact on the mental health of the population. Serafim and colleagues [7] found that nearly half of the participants in the Brazilian study reported symptoms of depression and stress. These findings are in line with the Campos et al. study [8] indicating that some demographic factors such as the female gender and low income can worsen the psychological symptoms reported by the participants. Another study, involving a sample of 1.996 Brazilian participants, found that somatic symptoms and sleep problems, along with depression, were the most frequently reported COVID-19-related symptoms [9]. Moreover, individuals who had a previous history of a mental disorder showed a higher severity of symptoms than those without. In any case, isolation due to restrictive measures is associated with depression and worsened overall mental health [10].

Likewise, there appears reason to assume that Italian people are affected by an analogous psychological impact. An alarming prevalence of depressive and stress-related symptoms was detected, corroborating the fact that the mental health consequences of the pandemic should be considered a public health concern by the health authorities. Not unexpectedly then, findings from an early cross-sectional study have depicted a relevant prevalence of individuals who reported psychological distress due to the pandemic [11]. Another study has documented that the COVID-19 pandemic is related to moderate-to-extremely severe psychological distress, which concerns about a third of the total sample, especially for individuals who have low psychological resilience [12]. Ultimately, studies on the Italian population converged in indicating that the COVID-19 pandemic had a strong psychological impact on the general population through a broad array of symptoms in both the first and second wave [13–16].

Additionally, studies conducted in the United States have depicted an alarming picture, even though the initial months of the pandemic were marked by only a slight adverse impact on mental health [17]. With few exceptions [18], results of longitudinal studies have suggested that mental health might worsen as the pandemic persists, especially during the most critical phases, and therefore, health authorities should constantly monitor psychological symptoms among the general population [19, 20]. As expected, since the prolonged restrictive measures following the pandemic, a significant effect in the United States was characterized by increased psychological distress [21, 22], which in turn has been suggested as a risk factor for substance use disorder [23]. Depressive symptoms and trauma-related symptomatology have been also reported, especially during the first months of the pandemic [24].

It appears the psychological impacts of COVID-19 and the related pandemic public health measures have varying impacts on mental health. However, no matter how the COVID-19 may impact a single country, the differences should continue to be considered on a larger scale to better determine future public health strategies. Although relatively limited, there is a growing literature on the comparison of mental health outcomes in individuals of different countries. A study of 78 countries and 18 languages has found that severe mental health problems due to COVID-19 are present in about 10 percent of the general population [25]. Another cross-sectional study of 8 countries has revealed that the general population of Poland, the Philippines, and Thailand reported the highest levels of depression and stress, whereas the Vietnamese reported the lowest [26, 27]. Interestingly, Chinese individuals had lower levels of stress and depression than Americans, though the former had higher levels of symptoms of acute traumatic stress. Nonetheless, more research is needed to deeply understand how the pandemic impacts the most affected countries in terms of mental health.

Psychological resilience has been identified as a factor crucially associated with individual mental health [28]. The definition of resilience is complex, since it refers to more than merely the absence of psychopathological conditions or the absence of adversities during the life course [29]. Such complexity is explained by the joint interaction of multiple biological, psychological, social, and ecological systems, in order to allow individuals to sustain, or improve their mental wellbeing when challenges occur [30]. Indeed, the definition of psychological resilience encompasses both capacities and processes able to promote adaptation to stressful or traumatic events [31, 32]. As Connor and Davidson have previously pointed, psychological resilience contributes to the individual positive response to negative events and adverse outcomes as a trait or as a state: trait resilience is configured as a long lasting and stable individual feature; conversely, state resilience is configured as a short-range factor, and therefore reactive to a recent event [33].

If on the one hand, as previously stated, the experience of the COVID-19 pandemic has been associated with an increase in psychological distress, on the other hand the protective role of resilience has emerged precisely during such a traumatic and stressful event. Accordingly, psychological resilience has been found inversely associated with depressive symptomatology in participants affected by the SARS-CoV-2 [34]. Moreover, the protective and positive role of psychological resilience has emerged among community population and health workers worldwide [35, 36], and it was a core topic of interest in Brazil [37], Italy [38, 39], and USA [40]. Specifically, psychological resilience has been suggested as a key positive factor able to promote individual adaptation and the population’s decrease of psychological distress in the United States [19]. In Brazil, the presence of depressive symptoms has been evidenced as a predictor of poor resilience; conversely, the positive and stimulating use of leisure time contributed to increased resilient responses to the pandemic [35]. Consistently, the adaptive role of psychological resilience was also confirmed in the Italian population, by considering psychological resilience one of the protective factors that might buffer the extent of distress resulting from the COVID-19 pandemic [41].

In line with these premises, the present study sought to investigate differences in post-traumatic stress and depressive symptoms in three severely affected countries during the pandemic, namely Brazil, Italy, and the United States; even though the pandemic has heavily affected each of these countries, we hypothesized a different psychological impact among them for the observed variables. Furthermore, the study aimed to investigate the contribution of psychological resilience in each country, in order to highlight potential different modalities, between the three countries, in individuals’ adaptation processes.

## Methods

### Design, participants, and inclusion/exclusion criteria

This cross-sectional study separately involved three national community populations, namely the Brazilian, the Italian, and the American population. We recruited participants aged 18 years or older.

### Procedures

Initially, the research protocols were submitted and approved by the respective research ethics committees in each country. Data collection was performed online. The instruments were implemented on Google Forms (Brazil and Italy) and SurveyMonkey (USA) platforms. Participants were invited to complete the study through social networks and research platforms (Facebook, Instagram, WhatsApp, Sona Systems). Once the participant clicked on the link to access the online form, a brief description of the study and the information sheet/consent was presented. Participants who voluntarily agreed to participate were taken to the sociodemographic questionnaire and other research instruments. Participants who did not accept to take part in the study were routed to a “thank you” page. Response time was approximately 15 min. Participants who were discarded from the further analysis were those whose compilation of the questionnaires contained missing data. The data were collected from February 2021 to June 2021, in each country involved in the study (i.e. Brazil, United States, and Italy).

### Measurements

A socio-demographic questionnaire prepared by the research team was used to collect demographic information, such as gender, age, educational level, and marital status. This questionnaire also collected information about the participants' experience during the COVID-19 pandemic.

Resilience was assessed using the 10-item Connor-Davidson Resilience Scale (CD-RISC-10) [42, 43]. The CD-RISC-10 is a self-administered questionnaire and has a single dimension. The scale has 10 items with five response options (0 = never; 4 = almost always). The abridged version of the CD-RISC reflects the ability to tolerate experiences such as change, personal problems, illness, pressure, failure, and painful feelings. The final questionnaire score is the sum of the responses to each item (range 0 to 40), where higher scores indicate greater resilience. The brief scale was developed by Campbell-Sills and Stein [44] by using factor analysis. They found a mean score of 31.8 (*SD* = 5.4) in a community sample of 764 US adults. These results have been replicated in other studies. The CD-RISC 10-item mean score was 32.1 ( *SD* = 5.8) in a USA national random digit dial sample [45]. A 2011 Brazilian study with a non-clinical sample of adults [46] yielded a mean score of 29.1 (*SD* = 5.5). In addition, the 10-item scale has shown extensive validation and has been found useful in many ways with many populations [47]. Cronbach’s alpha for the current study was 0.89 overall; 0.89 for Brazil, 0.91 for Italy, and 0.87 for the USA.

Depression was assessed using the scale developed by the Center for Epidemiological Studies Depression Scale (CES-D) for general population surveys [48, 49]. The 20-item scale assesses depressed mood, feelings of worthlessness, hopelessness and loneliness, loss of appetite, sleep disturbances, concentration problems, and psychomotor retardation. Respondents are asked to rate each symptom on a scale from 0 (rarely) to 3 (most or all of the time), according to how often they experienced the symptom during the past week. The scale score ranges from 0 to 60, with a higher score reflecting more depressive symptoms. Norming samples showed an internal consistency in the general population of about 0.85. Cronbach’s alpha for the current study was 0.92 overall and 0.94 in Brazil, 0.93 in Italy, and 0.81 in the USA.

Post-traumatic stress was assessed using the Impact of Event Scale Revised (IES-R) [50–52]. The IES-R is a self-administered instrument, in which the individual answers questions based on the 7 days prior to completion. The scale is composed of 22 items distributed in 3 subscales (avoidance, intrusion, and hyperstimulation). The score for each question ranges from 0 (not at all) to 4 (extremely). The total score is the sum of the subscale scores, with a higher score reflecting greater experience of post-traumatic symptoms in the previous week. The IES-R’s subscale coefficient alphas were found to be at 0.79 and above [52]. Cronbach’s alpha for the current study was higher than 0.84 overall and higher than 0.84 in Brazil, higher than 0.84 in Italy, and higher than 0.80 in the USA.

### Data analysis

Three separate Analyses of Covariance (ANCOVA) were conducted to examine whether there were differences in the levels of resilience, post-traumatic stress, and depression among the participants evaluated in Brazil, Italy, and the USA, while controlling for the effects of the covariates: age, gender, level of education, marital status, offspring, lost work or business during the pandemic, and tested positive for COVID-19. Data normality was assessed using the Shapiro–Wilk test. The assumption of homogeneity of variance was evaluated using Levene's test.

Bootstrapping procedures were performed (1000 re-samplings; 95% IC BCa) to obtain greater reliability of the results, to correct deviations from the normality of the sample distribution and differences between the sizes of the groups, and also to present an interval of 95% confidence for differences between means [53]. Post-hoc evaluations using the Bonferroni technique were requested [54].

## Results

A total of 734 participants participated in this study; 278 were from Brazil, 272 were from Italy, and 184 were from the United States. The total sample was composed of 576 participants identifying as female, 152 identifying as male, and 8 identifying as “other gender”. Age ranged from 18 to 69 years. A detailed description of the sociodemographic characteristics of each country is reported in Table 1.

**Table 1:** Sociodemographic characteristics of the three community populations

**Variable**	**Brazil**		**Italy**		**USA**		***p***
Age							
Mean (SD)	29.56 (10.23)		31.28 (12.65)		25.97 (11.78)		< 0.001^a^
Min—Max	18	66	18	69	18	68	
	***n***	***%***	***N***	***%***	***n***	***%***	
Gender							
Female	212	70.3%	208	75.9%	156	84.8%	
Male	64	29.0%	66	24.1%	22	12.0%	< 0.001^b^
Other	2	0.7%	0	0.0%	6	3.2%	
Level of Education							
Less than High School	2	0.7%	5	1.8%	3	1.6%	< 0.001^b^
High School	14	5.0%	44	16.1%	93	50.5%	
Undergraduate	185	66.5%	110	40.1%	74	40.2%	
Graduate Education	77	27.7%	115	42.0%	14	7.6%	
Marital Status							
Unmarried	185	66.5%	200	73.0%	146	79.3%	0.002^b^
Married	84	30.2%	62	22.6%	25	13.6%	
Widowed	1	0.4%	7	2.6%	2	1.1%	
Divorced	8	2.9%	5	1.8%	11	6.0%	
Offspring							
With Children	70	25.2%	67	24.5%	28	15.2%	0.025^b^
Without Children	208	74.8%	207	75.5%	156	84.8%	
Lost Work or Business during the pandemic							
No	245	88.1%	260	94.9%	152	82.6%	< 0.001^b^
Yes	33	11.9%	14	5.1%	32	17.4%	
Tested Positive for COVID-19							
No	221	79.5%	260	94.9%	142	77.2%	< 0.001^b^
Yes	57	20.5%	14	5.1%	42	22.8%	

^a^significance level of the Welch test for comparing means

^b^significance level of the Chi-square test of Pearson for comparing frequencies

The data obtained with measures of resilience, depression, and post-traumatic stress, obtained in the COVID-19 pandemic, were analyzed and the results of descriptive statistics, dispersion, and distribution are presented in Table 2. The normality distribution test demonstrated that, in the three variables described, at least one of the three groups is not normally distributed. Levene's test showed that there is no homogeneity of variance between the three groups, considering the variables resilience (Levene's *Z* (2, 673) = 7.522 *p* < 0.001), post-traumatic stress (Levene's *Z* (2, 730) = 5.479; *p* = 0.004), and depression (Levene's *Z* (2, 730) = 19.890; *p* < 0.001).

**Table 2:** Descriptive statistics for the evaluated psychological factors

	**CD_RISC-10**			**IES**			**CES-D**		
	**Brazil**	**Italy**	**USA**	**Brazil**	**Italy**	**USA**	**Brazil**	**Italy**	**USA**
Valid	278	274	125	278	274	185	279	274	185
Missing	0	0	59	0	0	0	0	0	0
Mean	22.90	25.24	25.19	32.72	33.06	21.13	24.28	22.08	22.62
Standard deviation	7.251	8.424	6.777	17.505	19.417	16.596	13.843	13.772	9.258
Shapiro–Wilk	0.993	0.972	0.990	0.981	0.969	0.935	0.981	0.953	0.972
*p* (Shapiro–Wilk)	0.208	< .001	0.478	< .001	< .001	< .001	< .001	< .001	< .001
Minimum	3.00	0.00	9.00	0.00	0.00	0.00	0.00	0.00	3.00
Maximum	40.00	40.00	40.00	76.00	88.00	68.00	57.00	54.00	47.00

*Abbreviations*: *CD_RISC-10* Connor-Davidson Resilience Scale, *IES* Impact of Event Scale, *CES-D* Center for Epidemiological Studies Depression Scale

In order to compare the averages obtained from the samples of the three countries in each variable, ANCOVA comparisons were performed to assess whether there are significant differences between the levels of resilience, post-traumatic stress, and depression, after controlling for the effects of the covariates: age, gender, level of education, marital status, offspring, lost work or business during the pandemic, and tested positive for COVID-19 (as presented in Table 1).

Regarding resilience, the ANCOVA results showed that the covariates age (*Z*(1, 667) = 0.009, *p* = 0.924; *η*^2^ = 0.000), lost work or business during the pandemic (*Z*(1, 667) = 9.771, *p* = 0.602; *η*^2^ = 0.000), and tested positive for COVID-19 (*Z*(1, 667) = 0.730, *p* = 0.393; *η*^2^ = 0.001) were not significant in the model and had a negligible effect. However, the covariates gender (*Z*(1, 667) = 19.300, *p* < 0.001; *η*^2^ = 0.028), level of education (*Z*(1, 667) = 5.202, *p* = 0.023; *η*^2^ = 0.008), and offspring (*Z*(1, 667) = 5.281, *p* = 0.022; *η*^2^ = 0.008) were significant in the model. Gender exhibited a moderate effect, while the level of education and offspring showed a small effect.

After controlling for the effects of all these covariates, the differences between the groups in the variables measuring resilience (*Z*(2, 667) = 9.771, *p* < 0.001; *η*^2^ = 0.028) also remained significant with a moderate effect.

Regarding post-traumatic stress, the ANCOVA results showed that the covariates age (*Z*(1, 724) = 0.284, *p* = 0.594; *η*^2^ = 0.000), level of education (*Z*(1, 724) = 0.929, *p* = 0.335; *η*^2^ = 0.001), and offspring (*Z*(1, 724) = 0.915, *p* = 0.339; *η*^2^ = 0.001) were not significant in the model and had a negligible effect.

However, the covariates gender (*Z*(1, 724) = 29.491, *p* < 0.001; *η*^2^ = 0.039), lost work or business during the pandemic (*Z*(1, 724) = 8.224, *p* = 0.004; *η*^2^ = 0.011), and tested positive for COVID-19 (*Z*(1, 724) = 5.616, *p* = 0.018; *η*^2^ = 0.008) were significant in the model. Gender and lost work or business during the pandemic exhibited a moderate effect, while being tested positive for COVID-19 showed a small effect.

After controlling for the effects of all these covariates, the differences between the groups in the variables measuring post-traumatic stress (*Z*(1, 724) = 36.421, *p* < 0.001; *η*^2^ = 0.091) also remained significant with a large effect.

Regarding depression, the ANCOVA results showed that the covariates age (*Z*(1, 724) = 3.821, *p* = 0.051; *η*^2^ = 0.005), level of education (*Z*(1, 724) = 0.890, *p* = 0.346; *η*^2^ = 0.001), offspring (*Z*(1, 724) = 0.928, *p* = 0.336; *η*^2^ = 0.001), and tested positive for COVID-19 (*Z*(1, 724) = 1.760; *p* = 0.185; *η*^2^ = 0.002) were not significant in the model and had a negligible effect.

However, the covariates gender (*Z*(1, 724) = 19.732, *p* < 0.001; *η*^2^ = 0.027) and lost work or business during the pandemic (*Z*(1, 724) = 5.497, *p* = 0.019; *η*^2^ = 0.008) were significant in the model. Gender exhibited a moderate effect, and lost work or business during the pandemic showed a small effect.

After controlling for the effects of all these covariates, the differences between the groups in the variables measuring depression (*Z*(1, 724) = 3.080, *p* < 0.047; *η*^2^ = 0.008) also remained significant with a small effect.

Table 3 shows the results of Bonferroni post-hoc tests, interpreted through bootstrapping procedures, which were performed for the variables resilience, post-traumatic stress, and depression. As for the resilience variable, significant differences were found between the groups from Brazil and Italy and between the groups from Brazil and the USA. The groups from Italy and USA were statistically similar. As for the post-traumatic stress variable, significant differences were found between the USA and Brazil groups and between the USA and Italy groups. The groups from Brazil and Italy were statistically similar. Regarding the depression variable, a significant difference was found between the Brazil and United States groups. However, the groups from Italy and the United States, as well as Brazil and Italy, were statistically similar.

**Table 3:** Bootstrapping (95% CI Bca) for test Bonferroni for multiple comparisons

Dependent Variable	Groups		Difference mean (I-J)	Bias	Standard Test Statistics	Bootstrap^a^			
						**Sig**		**Confidence interval 95%**	
								**Lower**	**Upper**
**CD-RISC-10**	Brazil	Italy	-2.424	0.005	0.659	0.002	^b^	-3.662	-1.084
	Brazil	USA	-3.032	0.022	0.814	0.001	^b^	-4.632	-1.346
	Italy	USA	-0.608	0.017	0.915	0.495		-2.467	1.148
**IES**	Brazil	Italy	-1.470	-0.005	1.557	0.346		-4.455	1.679
	Brazil	USA	13.391	0.121	1.772	0.001	^b^	9.271	17.398
	Italy	USA	14.861	0.126	1.904	0.001	^b^	10.679	18.806
**CES-D**	Brazil	Italy	1.510	-0.010	1.150	0.179		-0.665	3.825
	Brazil	USA	3.131	-0.043	1.140	0.006	^b^	0.905	5.306
	Italy	USA	1.621	-0.033	1.171	0.167		-0.678	3.855

*Abbreviations*: *CD_RISC-10* Connor-Davidson Resilience Scale, *IES* Impact of Event Scale, *CES-D* Center for Epidemiological Studies Depression Scale

^a^Based on 1000 bootstrap samples

^b^Significant differences.

Specifically, respondents in the USA and Italy reported significantly higher resilience scores than individuals in Brazil. For post-traumatic stress, the scores on the IES-R were relatively comparable for Brazil and Italy, and revealed greater experience of post-traumatic stress in contrast to the USA. Regarding depression, the respondents from Brazil exhibited significantly higher levels compared to the respondents from the United States. The group from Italy showed statistical similarity to both the Brazil and United States groups.

## Discussion

The current study explored the different psychological impacts of the restrictive measures during the COVID-19 pandemic in Brazil, Italy, and the United States, which denoted three countries differently, though severely, affected by the pandemic. We selected three indicators of resilience, depression, and post-traumatic stress, by using a cross-sectional design and three samples, one for each country. Indeed, a deep understanding of the psychological consequences on the general population appears paramount.

In their systematic review and meta-analysis examining the interplay between restrictive measures due to the pandemic and mental health, Prati and Mancini [4] found only a small effect size for depression and anxiety among the general population. However, several studies have found a worrisome prevalence of anxiety, stress, and depression since the pandemic has began [25].

First of all, we compared the averages obtained with the samples belonging to the three countries in regard to depression and post-traumatic stress symptoms. Because of its role in mitigating emotional distress during the pandemic [12, 55], we also included psychological resilience in our survey.

As revealed by our results, after controlling for covariates, there were significant differences between Brazil, Italy, and the USA for the depression reported by the participants. Indeed, Brazilian participants experienced higher depressive symptoms than those from the USA. In spite of these differences, literature showed conflicting evidence about depression. A certain heterogeneity in depressive symptoms has been reported in a recent meta-analysis conducted by Bueno-Notivol and colleagues, which included twelve studies and six countries (i.e. China, Vietnam, India, Italy, United Kingdom, and Denmark) [56], with a pooled prevalence of depressive symptoms of 25%, despite considerable heterogeneity between studies especially due to the different self-reported instruments used [56]. Our findings seem to be consistent with this kind of literature.

Like the symptoms of depression, our findings indicated that there were significant differences between the three countries for psychological resilience and post-traumatic stress. This evidence might be explained by the reactive nature of these three constructs, which therefore makes them more sensitive to individual differences. A flourishing literature has focused on post-traumatic stress symptoms during the pandemic in both infected patients and healthcare workers [57, 58]. By the same token, several researchers have turned attention to the general population [59, 60], even though very few studies have compared these symptoms among different countries. The interplay between post-traumatic stress symptoms and resilience represents another fundamental caveat for understanding individual reactions to the pandemic. Available data have hitherto indicated that resilience mitigates the impact of pandemic [12, 61], though this conclusion requires few comments. A study comparing resilience in adults from Israel, the Philippines, and Brazil found that the latter showed the lowest level scores [62]. Most notably, the obtained scores were lower than the published normative data for this scale [33]. Results of this kind were found in other countries. Indeed, another study, involving a sample of 1,004 US adults during the lockdown, found lower levels of resilience than normative data [63]. In this vein, our findings may increase the understanding of the impact of the pandemic across countries. In our opinion, more attention to the underlying psychological factors of these symptoms, such as resilience, would constitute a promising tool for a preventive approach. Related, we found significant differences in resilience among the three countries. Still, it is worth emphasizing that high resilience during the COVID-19 pandemic contributed to decreased levels of depression and anxiety, but not post-traumatic stress symptoms [64].

In this regard, our findings underlining significant differences in resilience among Brazil, Italy, and the USA is partially coherent with what has been found by Prati and Mancini [4]. They argued that the majority of individuals showed resilience in the face of an ongoing pandemic, even though great heterogeneity was present. This is not surprising as the literature on potentially traumatic events has well established that most individuals show a trajectory of resilience [65]. Likewise, lockdown consequent to the pandemic may not have had uniform detrimental effects on mental health in the general population, although it is necessary to grasp the differences between countries, especially when they have been hit hard, such as Brazil, Italy, and the United States. Therefore, we carried out a series of post-hoc tests for resilience and post-traumatic stress. With regard to resilience, our results indicated significant differences between Brazil and Italy as well as between Brazil and the United States. Simply put, Italian and U.S. participants displayed similar and higher levels of resilience than those from Brazil. It is worthwhile to underscore that the mean scores of resilience exhibited by our sample of USA participants were analogous to the sample of Liu et al. [64].

As previously stated, our findings showed significant differences in post-traumatic stress symptoms among the three countries insofar as Brazilian and Italian participants had higher levels of post-traumatic stress symptoms than USA participants. This finding appears consistent with Wand and colleagues’ study [66] insofar as they depicted that USA participants seem to show fewer symptoms than other populations such as Chinese individuals.

The findings of this multi-national study may have relevant implications for public mental health policy. The first implication regards the possibility to implement specific prevention interventions aiming to decrease the psychological impact of the pandemic across different countries. Another implication concerns the possibility to implement psychological interventions for individuals that demonstrate major symptoms of mental disorders.

Although our findings add evidence to the understanding of the psychological impact of the COVID-19 pandemic in Brazil, Italy, and the USA, there are also some limitations that future studies should take into account. First, the cross-sectional design did not allow us to determine causal relationships among the observed variables. Second, the oversampling of certain characteristics such as gender may influence the results we obtained. Third, data were collected through an online survey that did not allow us to exclude pre-existing psychiatric disorders among respondents with absolute certainty. Ultimately, the use of self-report questionnaires and scales should be accounted as a limitation, since it did not allow proper control of the quality/accurateness of the participants’ responses.

## Conclusions

Overall, our results suggested significant differences between Brazil, Italy, and the USA. for resilience, post-traumatic stress symptoms, and depression. Specifically, Italian and USA participants showed higher levels of resilience than those from Brazil. Another relevant finding of this study was that Brazilian and Italian participants demonstrated higher levels of post-traumatic stress symptoms than those from the USA. Finally, Brazilian participants reported higher depressive symptoms than those form the USA.

Taken together, the results of this study give us a glimpse into the specific psychological impact of the pandemic among three of the most hit nations: Brazil, Italy, and the USA. Further research into the distinctions and similarities seems important, especially as we look to interventions and moving forward.

## Data Availability

The datasets used and/or analysed during the current study are available from the corresponding author on reasonable request.
